# HIV and chronic kidney disease 

**DOI:** 10.5414/CNP83S032

**Published:** 2015-02-23

**Authors:** Saraladevi Naicker, Sadaf Rahmania, Jeffrey B. Kopp

**Affiliations:** 1 Division of Nephrology, Department of Internal Medicine, School of Clinical Medicine, Faculty of Health Sciences, University of Witwatersrand, Johannesburg, South Africa, and; 2 Kidney Disease Section, National Institute of Diabetes and Digestive and Kidney Diseases, National Institutes of Health, Bethesda, MD, USA

**Keywords:** human immunodeficiency virus, kidney disease, risk factors, screening

## Abstract

Chronic kidney disease (CKD) is a frequent complication of HIV infection, occurring in 3.5 – 48.5%, and occurs as a complication of HIV infection, other co-morbid disease and infections and as a consequence of therapy of HIV infection and its complications. The classic involvement of the kidney by HIV infection is HIV-associated nephropathy (HIVAN), occurring typically in young adults of African ancestry with advanced HIV disease in association with APOL1 high-risk variants. HIV-immune complex disease is the second most common diagnosis obtained from biopsies of patients with HIV-CKD. CKD is mediated by factors related to the virus, host genetic predisposition and environmental factors. The host response to HIV infection may influence disease phenotype through activation of cytokine pathways. With the introduction of antiretroviral therapy (ART), there has been a decline in the incidence of HIVAN, with an increasing prevalence of focal segmental glomerulosclerosis. Several studies have demonstrated the overall improvement in kidney function when initiating ART for HIV CKD. Progression to end stage kidney disease has been reported to be more likely when high grade proteinuria, severely reduced eGFR, hepatitis B and/C co-infection, diabetes mellitus, extensive glomerulosclerosis, and chronic interstitial fibrosis are present. Improved renal survival is associated with use of renin angiotensin system blockers and viral suppression. Many antiretroviral medications are partially or completely eliminated by the kidney and require dose adjustment in CKD. Certain drug classes, such as the protease inhibitors and the non-nucleoside reverse transcriptase inhibitors, are metabolized by the liver and do not require dose adjustment. HIV-infected patients requiring either hemo- or peritoneal dialysis, who are stable on ART, are achieving survival rates comparable to those of dialysis patients without HIV infection. Kidney transplantation has been performed successfully in HIV-infected patients; graft and patient survival appears to be similar to that of HIV-uninfected recipients. Early detection of kidney disease by implementation of screening on diagnosis of HIV infection and annual screening thereafter will have an impact on the burden of disease, together with access to ART to those who require it. Programs for prevention of HIV infection are essential to prevent this lethal disease.

## Introduction 

The majority of the 35.3 million people living with HIV worldwide reside in sub-Saharan Africa, an estimated 24.7 million people [1]. An estimated 9.7 million people in low- and middle-income countries were receiving antiretroviral therapy (ART) in December 2012, representing 34% of the estimated 28.6 million people eligible, with 875,000 people receiving ART in high-income countries. 

Kidney disease is a frequent complication of HIV infection, occurring in 3.5 – 48.5% [2], and occurs as a complication of HIV infection, other co-morbid disease, and infections and as a consequence of therapy of HIV infection and its complications. We therefore reviewed the patterns of chronic kidney disease (CKD), its risk factors, and outcomes in individuals with HIV infection. 

## Patterns of CKD in HIV infection 

The classic involvement of the kidney by HIV infection is HIV-associated nephropathy (HIVAN), which was originally reported in New York in 1984, and clinically presents with proteinuria and renal dysfunction and pathologically with focal segmental glomerulosclerosis (FSGS) with collapsing glomerulopathy, microcystic tubular dilatation, and interstitial inflammation [3]. It has been reported in 3.5 – 10% of the HIV-infected population in the USA, predominantly in individuals of African ancestry. HIVAN has been described typically in young adults of African ancestry with advanced HIV disease, though it may occasionally be diagnosed before acute HIV seroconversion has been identified [4] and presents with nephrotic-range proteinuria and rapid progression to end-stage renal disease (ESRD) without ART. 

HIV-immune complex disease (HIV-ICD) has been reported predominantly in people of European and Asian ethnicity, but has also subsequently been reported in people of African origin [5, 6]. 

HIV-immune complex disease is the second most common diagnosis obtained from biopsies of patients with CKD. Gerntholtz et al. [5] reported a biopsy series in 99 HIV patients with renal insufficiency in which 21% had HIV-ICD with the largest group being HIVAN at 27%. Another South African study of 221 patients biopsied in Cape Town showed 26% with features of HIV-ICD [6]. The clinical profile of the patients was similar to that of HIVAN, although they had less proteinuria and better serum creatinine and albumin levels. 

HIV-ICD presents with a variety of histological changes (Table 1). Histologically, the predominant pattern is variable mesangial alteration with immune deposits in the mesangial and paramesangial regions. Subepithelial immune deposits are also seen and described as a “ball-in-cup” basement membrane reaction [5]. Other forms of HIV-ICD include membranoproliferative glomerulonephritis, membranous nephropathy, and post-infectious glomerulonephritis. These are often seen in patients who are co-infected with hepatitis B and/or C viral infection. In the study by Gerntholtz et al. [5], of the 99 patients who were biopsied 13% had membranous nephropathy, 8% post-infectious glomerulonephritis (GN) and 6% membranoproliferative GN. It is not known if the pathogenesis of these is directly related to HIV infection. Explanations could include abnormal immune responses associated with viral infection, or responses secondary to super-infections. 

## Pathogenesis of CKD in HIV infection 

CKD is mediated by factors related to the virus host, genetic predisposition, and environmental factors. 

The question of whether HIV directly infects renal cells is an issue central to pathogenesis. Due to the lack of CD4 and chemokine receptors needed for entry into cells, viral replication is likely restricted. Evidence from transgenic mouse models suggest that expression of single HIV genes can replicate the clinical features (proteinuria, progessive kidney disease) and pathologic features (collapsing glomerulopathy, tubular cell injury) of HIVAN as seen in human patients. Transfection of viral constructs allows renal epithelial cells to produce viral products. Cells transfected with CD4 and CXCR4 chemokine receptors support viral replication. It is still unclear how HIV-1 enters renal cells [7]. Genetic variability of gp120 seems to influence renal infectivity. Lymphocytes may allow cell infection in a monolayer via transcytosis. Another possible mechanism is transfer of CCR5 between cells (these contain cell surface and cytoplasmic components of the original cell), thus allowing entry of the HIV virus into renal cells without endogenous expression of the co-receptor. Dendritic cells have been found to be involved in binding, dissemination and transfer of HIV in a variety of tissues and may also play a role in infection of renal cells. The dendritic cell C-type lectin receptor DEC-205 has been shown to mediate internalization of HIV into human renal tubular cells. There is increasing evidence to suggest that renal cells may support viral replication [8]. 

### Viral proteins 

Transgenic mice that express particular combinations of viral proteins have been used as a model for renal pathogenesis. Studies have suggested that *vpr* may play a role in the development of FSGS, and macrophage-specific expression of HIV proteins may be important. Others report that *nef* may contribute to the severity of interstitial nephritis and the glomerular changes seen in HIVAN [9]. Podocyte-restricted expression of *vif, nef, tat, vpr*, and *rev* have been shown to induce many of the features of HIVAN in mice models [10]. Another feature of HIV-1 infection is apoptosis of renal epithelial cells mediated by *Fas* up-regulation and caspase activation. This has been seen in HIVAN specimens [7]. 

### Host factors 

Individuals of African descent are predisposed to HIVAN. Genetic variants of recent African origin might account for this susceptibility and was mapped by admixture linkage dysequilibrium (MALD). A locus on chromosome 22 was found to have a strong association with HIV kidney disease in African-Americans [11]. Variants in *APOL1*, encoding apolipoprotein L1, accounted for most of this increased risk [12]. These variants include two missense mutations (S342G, Rs73885319 and I384M, Rs60910145) that are in close linkage dysequilibrium, termed G1, and a two base pair deletion (N388del, Y389del, Rs71785313), termed G2. The G1 and G2 variants confer risk for HIVAN and HIV-associated FSGS, as well as other glomerular disease and hypertensive nephrosclerosis. 

The published odds ratios for the recessive model are 29 for HIVAN and 17 for primary FSGS [13]. In unpublished work, we have found that the odds ratio for HIVAN is numerically higher in South Africa (89, CI 18 – 911) [Kasembeli, in press]. We estimate that 50% of HIV-positive individuals who have two *APOL1* risk alleles and do not receive effective ART will develop HIVAN, indicating a powerful genetic propensity. The mechanisms by which the APOL1 variants alter kidney cell function is a matter of considerable interest. 

It has been suggested that the host response to HIV infection may influence disease phenotype through activation of cytokine pathways. It has been shown that multiple mediators of the inflammatory response including cytokines, chemokines, and adhesion molecules are up-regulated in renal epithelial cells of patients with HIV-associated renal disease. Many of these up-regulated genes are targets of NFκ-B and IL-6. TNF and IL-6 expression by mesangial and tubular epithelial cells stimulate HIV-1 expression by infiltrating monocytes and further drive cytokine production. The role of inflammatory mediators in the pathogenesis of HIVAN is not yet entirely understood [14]. 

Chronic HIV infection is associated with polyclonal expansion of immunoglubulins. Immune complexes that circulate in the systemic circulation may be deposited in the renal microcirculation, giving rise to HIV immune complex kidney diseases [15]. 

The renal infiltrate in HIV-ICD consists primarily of B lymphocytes, in contrast to HIVAN where it is composed mainly of T lymphocytes and macrophages [16]. The pathogenesis is thought to be associated with the development of polyclonal hypergammaglobulinemia, thus promoting the circulation of immune complexes which are then passively trapped in the kidney. Activation of inflammatory mediators subsequently occurs which then results in secondary renal damage similar to that of lupus nephritis. Another mechanism could also be the in-situ deposition of antibodies binding to HIV viral antigens within the kidney [16]. 

## Impact of antiretroviral therapy on CKD 

Prior to the availability of cART, HIVAN almost uniformly progressed rapidly to ESRD. With the introduction of cART, there has been a decline in the incidence of HIVAN in the USA [17, 18]. HIVAN risk was reduced by 60% with the use of cART. A recent study from France described the change in the pattern of renal disease in HIV patients over 15 years since the introduction of ART; HIVAN decreased over the 15 years and classic FSGS emerged as the commonest cause of glomerular disease during 2004 – 2007, occurring in 46.9% [19]. HIVAN occurred more frequently in Black patients with severe immunodeficiency and severe renal failure in this study, while FSGS patients were older, more likely to have received ART and more frequently had cardiovascular risk factors and histologically, had more severe interstitial fibrosis. 

Older guidelines recommend HIVAN as an indication for the initiation of cART, irrespective of the CD4 lymphocyte count [20]. Current HIV guidelines recommend use of cART in all patients with HIV; resource constraints in some regions of the world, for example South Africa recommend cART initiation with CD4 < 350 cells/mL. Epidemiologic data showing the decline in HIVAN and HIV-associated ESKD in the United States after the introduction of ART in 1995 suggest that effective control of viral replication with ART can prevent the appearance of HIVAN. While the evidence for initiating cART in HIV-ICD is inconclusive, this seems to be a feasible approach. 

There have been conflicting reports on the benefit of cART in patients with HIV-ICD. A study done by Szczech et al. [21] found that renal function in patients with lesions other than HIVAN, including immune complex kidney disease did not benefit from ARTs. Two studies in South Africa showed improvement in renal function with cART irrespective of renal histology [6, 22]. Wearne et al. [6] in South Africa reported that both HIVAN and HIV-ICD showed response to cART. 

Immunosuppressive therapies, such as corticosteroids, to dampen the inflammatory response to these complexes at the level of the kidney have been suggested as possible additional strategies for treatment. 

Several studies have demonstrated the overall improvement in kidney function when initiating cART for HIV CKD. The DART study conducted in Uganda and Zimbabwe, showed improvement of GFR by 1.9 – 6 mL/min/ 1.73 m^2^ after 4 – 5 years of ART, with 2.8% of the 3,316 patients at an eGFR < 30 mL/min/ 1.73 m^2^ [23]. An improvement in median eGFR by 21% after 2 years on cART was reported in Ugandan patients with HIV CKD [24]. A recent study from Tanzania showed improvement in renal function on ART over a median period of 2 years, with the numbers of patients with eGFR < 90 mL/min/1.73 m^2^ decreasing from 76% to 29.2% and those with eGFR < 60 mL/min decreasing from 21.1% to 1.1% [25]. 

Many antiretroviral medications are partially or completely eliminated by the kidney and require dose adjustment in CKD. Certain drug classes, such as the protease inhibitors and the non-nucleoside reverse transcriptase inhibitors (NNRTIs), are metabolized by the liver and do not require dose adjustment [26]. Most of the nucleoside reverse transcriptase inhibitors (NRTIs) are excreted unchanged in the urine and require dose adjustment. The NRTI dose may have to be supplemented following dialysis [27]. Fixed drug combinations should not be used in patients with eGFR below 30 to 50 mL/min/1.73 m^2^. 

## Risk factors for developing CKD and ESRD 

Several studies have pointed to HIV infection being an independent risk factor for microalbuminuria. A study done in the United States showed that 11% of HIV-positive patients had microalbuminuria. It was found that the odds were 5 times higher for those with HIV to have microalbuminuria than control patients. Predictors for albuminuria in HIV patients included lower CD4 count, higher viral load, and African-American race [28]. In another study, older age, black race, hepatitis C infection, and lower CD4 count were independently associated with CKD. Of note, virological suppression was also more common with renal impairment, most likely due to higher blood levels of renal-eliminated ARTs [29]. More recently, a study involving multiple urine collections found the period prevalence of HIV microalbuminuria to be 14%, with single collection giving a positive predictive value of 74% (suggesting that microalbuminuria is sometimes transient) and a negative predictive value of 98% (suggesting that annual screening is sufficient to detect persistent albuminuria) [30]. 

Progression to ESRD has been reported to be more likely when the following parameters are present: high-grade proteinuria, severely reduced eGFR, hepatis B and/C co-infection, diabetes mellitus, extensive glomerulosclerosis, and chronic interstitial fibrosis [31, 32]. Improved renal survival was associated with use of renin angiotensin system blockers and viral suppression. HIVAN patients with two APOL1 high renal variants progressed more rapidly to ESRD in spite of effective viral suppression [33]. 

## Renal replacement therapy 

HIV-infected patients requiring either hemo- or peritoneal dialysis, who are stable on ART, are achieving survival rates comparable to those of dialysis patients without HIV infection, and choice of dialysis modality does not have an impact on survival. Strict adherence to universal precautions is the best form of prevention of HIV transmission in dialysis units. 

Kidney transplantation has been performed successfully in HIV-positive patients. A series of 150 kidney transplants in the USA has reported patient and graft survival of 88.2% and 73.7%, respectively, at 3 years. In spite of high rates of acute graft rejection, graft and patient survival appears to be similar to that of HIV-uninfected recipients [34]. 

## Screening, early diagnosis, and therapy of HIV CKD 

Patients with HIV disease are at increased risk for CKD and should receive regular screening for renal disease. The Infectious Diseases Society of America has proposed that annual screening should include assessment of blood pressure, serum creatinine and/or cystatin C, and a quantitative measure of urine protein [20]. When a low albumin/protein ratio suggests proximal tubular disease, this can be confirmed by measuring the β2 microglobulin/creatinine ratio, as well as by demonstrating increased excretion of phosphate and urate. 

Several therapies were attempted for HIVAN in the early years of the HIV epidemic. Several studies in the pre-ART era have suggested a benefit from corticosteroid therapy, but because the efficacy appears to be modest and often short-lived, this was not considered standard therapy. Similarly, there was limited data on the efficacy of cyclosporine in inducing remission of proteinuria in children with HIVAN [35]. The HIV Medicine Association of the Infectious Diseases Society of America recommends that all patients with HIVAN should receive ART; where renal function does not improve, angiotensin converting enzyme inhibitors or angiotensin receptor blockers and/or prednisone (dose of 1 mg/kg/day; maximum dose 80 mg/day for 2 months, with tapering over 2 – 4 months) may be added [20]. 

Standard therapies for CKD are recommended, including control of blood pressure to below 140/90 mmHg and the use of angiotensin-converting enzyme (ACE) inhibitors or angiotensin receptor blockers (ARBs). Underdosing of ART in CKD has been reported to be associated with increased mortality [36, 37]. General therapies include control of serum urate and bicarbonate, avoidance of nephrotoxins, smoking cessation, and weight loss in obese patients [38]. 

The NRTIs are primarily excreted by the kidneys; therefore reduced dosages are required for those with impaired renal function, with GFRs < 60 mL/min. Patients who have then been identified with CKD are recommended to have more frequent monitoring of kidney function, toxicity, and therapeutic efficacy. Care should be taken not to allow for underdosing as this may lead to inadequate treatment and contribute to virological failure. The NRTIs are not tightly protein-bound and low to medium molecular weight and therefore can be easily removed by dialysis. With the exception of abacavir, the NRTIs should generally be administered after dialysis. With the exception of nevirapine and indinavir, the NNRTIs, protease inhibitors (PIs) and fusion inhibitors are primarily excreted by the liver and do not need dose adjustment; these two should be administered after dialysis [34]. 

## Conclusion 

Together with increasing life expectancy in patients receiving cART, ageing of the HIV-infected population and drug nephrotoxicity, the burden of CKD is escalating. Early detection of kidney disease by implementation of screening on the diagnosis of HIV infection and annual screening thereafter will have an impact on the burden of disease, together with access to cART to those who require it. Programs for prevention of HIV infection are essential to prevent this disease that is lethal without treatment and is associated with prolonged survival with cART. 

## Acknowledgment 

The Medical Research Council of South Africa and the National Research Foundation of South Africa for funding. The NIDDK Intramural Research Program. 

**Table 1.** Spectrum of renal lesions in HIV infection.

**Kidney disorder**	Associations/subtypes
**Tubulo-interstitial disease**	
Acute kidney injury	Sepsis, toxins, drugs
Proximal tubular injury	Tenofovir, adefovir, codofovir, didanosine
Chronic tubular injury	Amphotericin, tenofovir, adefovir, cidofovir
Crystal nephropathy	Indinavir, atazanavir, sulphadiazine, ciprofloxacin, acyclovir (IV)
Interstitial nephritis	Infections (including HIV, BK virus), following ART, drugs
**Glomerular lesion**	
HIV-FSGS (focal segmental glomerulosclerosis) or “classic” HIVAN (HIV-associated nephropathy: FSGS with collapsing glomerulopathy, microcystic tubular dilatation, interstitial inflammation)	APOL1 risk variants
HIV-ICD (HIV-immune complex disease) (this group may have co-infection with Hepatitis B or C)	Mesangial proliferative Membranoproliferative (types I and III) Lupus-like Exudative-proliferative Crescentic IgA Membranous
Various glomerulonephropathies (this is a heterogeneous group with different etiologies)	Minimal change disease Membranous nephropathy Immunotactoid nephropathy Amyloidosis
HIV-TTP/HUS	TTP: Thrombotic thrombocytopenic purpura HUS: Hemolytic uremic syndrome
